# Parkin facilitates proteasome inhibitor-induced apoptosis via suppression of NF-κB activity in hepatocellular carcinoma

**DOI:** 10.1038/s41419-019-1881-x

**Published:** 2019-09-26

**Authors:** Xiaolan Zhang, Chun Lin, Junwei Song, Han Chen, Xuhong Chen, Liangliang Ren, Zhongqiu Zhou, Jinyuan Pan, Zhenjun Yang, Wenhao Bao, Xueping Ke, Jianan Yang, Yingying Liang, Hongbiao Huang, Daolin Tang, Lili Jiang, Jinbao Liu

**Affiliations:** 1 Affiliated Cancer Hospital & Institute of Guangzhou Medical University, Key Laboratory of Protein Modification and Degradation, State Key Laboratory of Respiratory Disease, School of Basic Medical Sciences, Guangzhou Medical University, 511436 Guangzhou, China; 2 Guangdong Key Laboratory for Genome Stability and Human Disease Prevention, Department of Biochemistry and Molecular Biology, Health Science Center, Shenzhen University, 518060 Shenzhen, China; 3 Department of Biochemistry, Zhongshan School of Medicine, Sun Yat-sen University, 510080 Guangzhou, China; 4 Department of Gastroenterology, the Liwan Hospital of the Third Affiliated Hospital of Guangzhou Medical University, 510175 Guangzhou, China; 5 Department of Urologic Oncosurgery, Affiliated Cancer Hospital & Institute of Guangzhou Medical University, 510095 Guangzhou, China; 6 Department of Ultrasonography, Guangzhou Women and Children’s Medical Center, Guangzhou Medical University, 510623 Guangzhou, China; 7 Department of Surgery, UT Southwestern Medical Center, Dallas, TX 75390 USA

**Keywords:** Cancer, Cell biology

## Abstract

The ubiquitin–proteasome system (UPS) is a tight homeostatic control mechanism of intracellular protein degradation and turnover involved in many human diseases. Proteasome inhibitors were initially developed as anticancer agents with potential benefits in the suppression of tumor growth. However, clinical trials of patients with solid tumors fail to demonstrate the same efficacy of these proteasome inhibitors. Here, we show that Parkin, an E3 ubiquitin ligase, is implicated in tumorigenesis and therapy resistance of hepatocellular carcinoma (HCC), the most common type of primary liver cancer in adults. Lower *Parkin* expression correlates with poor survival in patients with HCC. Ectopic *Parkin* expression enhances proteasome inhibitor-induced apoptosis and tumor suppression in HCC cells in vitro and in vivo. In contrast, knockdown of *Parkin* expression promotes apoptosis resistance and tumor growth. Mechanistically, *Parkin* promotes TNF receptor-associated factor (TRAF) 2 and TRAF6 degradation and thus facilitates nuclear factor-kappa-B (NF-κB) inhibition, which finally results in apoptosis. These findings reveal a direct molecular link between Parkin and protein degradation in the control of the NF-κB pathway and may provide a novel UPS-dependent strategy for the treatment of HCC by induction of apoptosis.

## Introduction

The intracellular proteins are continually turning over, which is controlled by synthesis and degeradation. The ubiquitin–proteasome system (UPS) is a part of the protein degradation system, whereas ubiquitinylation is a form of post-translational modification. UPS plays a key role in the protein degradation and turnover involved in multiple physiological and pathological processes, including cell survival and cell death^1^. Deregulation of the UPS is implicated in tumor initiation and development partly through the downregulation of tumor-suppressor proteins or upregulation of oncogenic proteins^2,3^. Proteasome inhibitors were initially developed as anticancer agents with potential benefits in preventing tumor growth^3^. The first-generation proteasome inhibitor such as bortezomib (also known as PS341) and the second-generation proteasome inhibitor, such as including carfilzomib, ixazomib, and oprozomib, have been demonstrated to improve the clinical outcomes in certain hematological malignancies, including acute myeloid leukemia, myelodysplastic syndrome, and acute lymphoblastic leukemia^4–7^. Despite the promising clinical activity in hematological malignancies, clinical trials of patients with solid tumors failed to demonstrate the same efficacy of these proteasome inhibitors, especially bortezomib^8^. The basis for bortezomib resistance can be through multiple pathways^9–11^. For example, the pharmacokinetic and pharmacodynamic characteristics of bortezomib may impair the distribution of bortezomib into solid tumors. A higher dose of bortezomib also displays significant toxic effects which limit its utilization in the treatment of patients with solid tumors^8,12^.

Constitutive activation of the transcription factor NF-κB signaling is a hallmark of cancer^13^. NF-κB regulates multiple aspects of tumor biology and mediates survival and therapy resistance through inducing the expression of cytokines, antiapoptotic factors, and adhesion molecules^14,15^. Under normal conditons, NF-κB locates in the cytosol due to binding to its inhibitor—inhibitor of NF-κB (IκB). IκB can be phosphorylated by the inhibitor of nuclear factor-kappa-B kinase (IKK) complex, and then ubiquitinated and degraded through the UPS. Loss of IκB initiates NF-κB translocation from the cytosol to the nucleus where it triggers gene transcription^16,17^. Thus, the original idea was proposed to suppress tumor growth by inhibition of NF-κB signaling via blocking IκB degradation^18–20^. However, prevention of IκB degradation by the IKK inhibitor only results in a 20–50% decrease in cell proliferation^21^, suggesting that the IκB-independent pathway also contributes to the anticancer activity of proteasome inhibitors.

Parkin mutations are the most common genetic cause of the early onset of Parkinson’s disease (ARPD)^22^. As a E3 ubiquitin ligase, parkin mutations cause protein accumulation and subsequent neural cell death in Parkinson’s disease (PD)^23^. In addition to PD, parkin mutations are also implicated in cancer. *Parkin* is localized to the human chromosome 6q25–27, a region frequently lost in cancers. Indeed, loss of heterozygosity and copy number of *Parkin* has been observed in many types of cancers, such as breast, lung, colorectal, and ovarian cancers, hepatocellular carcinoma, non-small-cell lung carcinoma, and lymphomas^24–26^. As a tumor suppressor, Parkin can induce cell cycle arrest in G1/S and inhibit cell proliferation through degradation of cyclin E or cyclin D in glioma^27,28^. Lower Parkin expression correlates with poorer distant metastasis-free survival in breast cancer and Parkin suppresses metastasis through degradation of HIF-1α^29^. Parkin-mediated HIF-1α degradation or p53 inhibiton is also involved in the regulation of metabolic reprogramming during breast cancer and glioma progression^29–31^. In addition, Parkin suppresses pancreatic tumorigenesis through control of the mitochondria turnover and the subsequent mitochondrial iron-mediated immunometabolism^32^. Collectively, these findings suggest that Parkin is a potential tumor suppressor. However, the dysfunction of the Parkin pathway in cancer has not been fully elucidated.

In the present study, we found that lower *Parkin* expression correlates with poor survival in patients with HCC, the most common type of primary liver cancer in adults. Importantly, we demonstrated that Parkin promotes anticancer activity of the proteasome inhibitor through inhibition of NF-κB via direct degradation of TRAF2 and TRAF6 in HCC cells. These findings not only suggested a new mechanism of Parkin-mediated apoptosis, but also provided a novel strategy for the overcoming of drug resistance of the proteasome inhibitor.

## Results

### Parkin is downregulated in HCC

**Fig. 1:**
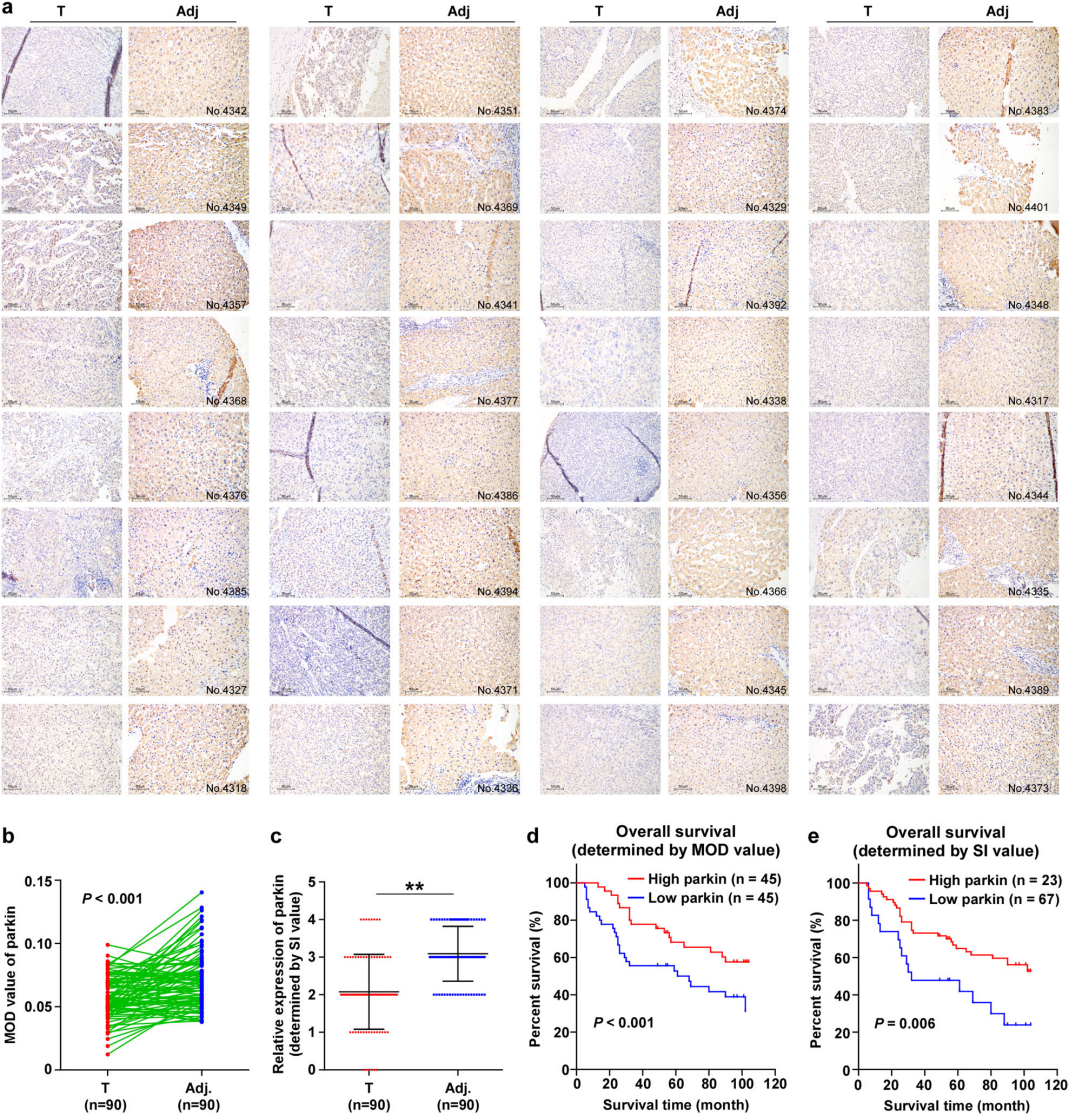
Parkin is downregulated in HCC. **a** IHC analysis of Parkin expression in formalin-fixed paraffin-embedded HCC tissues (T), compared with the adjacent non-tumor tissues (Adj.) from the same patients. **b** Quantification of IHC analysis of Parkin expression. The MOD value was used for the quantification of IHC analysis and *Image J pro* software was used to determine the MOD value. **c** The staining index (SI) was used for the quantification of IHC staining. **P < 0.01. **d** and **e** Kaplan–Meier analysis of overall survival for patients with HCC stratified by low versus high expression of Parkin, determined by the MOD value (**d**) and SI score (**e**), respectively. *P* values were calculated by using the log-rank test

A tissue array (No. *HLivH180Su14*), including 90 pairs of clinical HCC samples associated with their paired adjacent non-tumor tissues, was used for the evaluation of parkin expression by IHC staining. The expression of Parkin was significant in normal hepatic tissues, while it was downregulated in the samples from paired HCC tissues (Figs. 1a–c and S1a). Kaplan–Meier analysis and log-rank testing revealed that the lower Parkin expression correlated with a shorter survival time, whereas the higher Parkin expression correlated with a longer survival time (*P* *<* 0.01; Fig. 1d, e), which was consistent with the result of KM-plotter data analysis (*P* = 0.024, Fig. S1b). Thus, downregulation of Parkin correlates with poorer survival of HCC patients, indicating that Parkin is a potential tumor suppressor during HCC progression.

To further investigate the role of Parkin in HCC, we examined the level of Parkin in HCC cell lines and normal liver cells. Western blot and Q-PCR analysis showed that both protein and mRNA expression of Parkin were significantly lower in the HCC cell lines compared with the normal LO2 human liver cells (Fig. S1c). Analysis of *Parkin* copy-number variation (CNV) by using the liver hepatocellular carcinoma (LIHC) dataset from The Cancer Genome Atlas (TCGA) showed that the *Parkin* locus was deleted in 38.4% HCC samples and that *Parkin* expression was significantly associated with *Parkin* CNV (Fig. S2a, b). Moreover, analysis of TCGA datasets also revealed that both the *Parkin* expression and CNV were downregulated in the subsets of many tumors (Fig. S2c, d). These results support that Parkin is a tumor suppressor in multiple types of cancers.

### Parkin facilitates the PS341-induced apoptosis of HCC in vivo

**Fig. 2:**
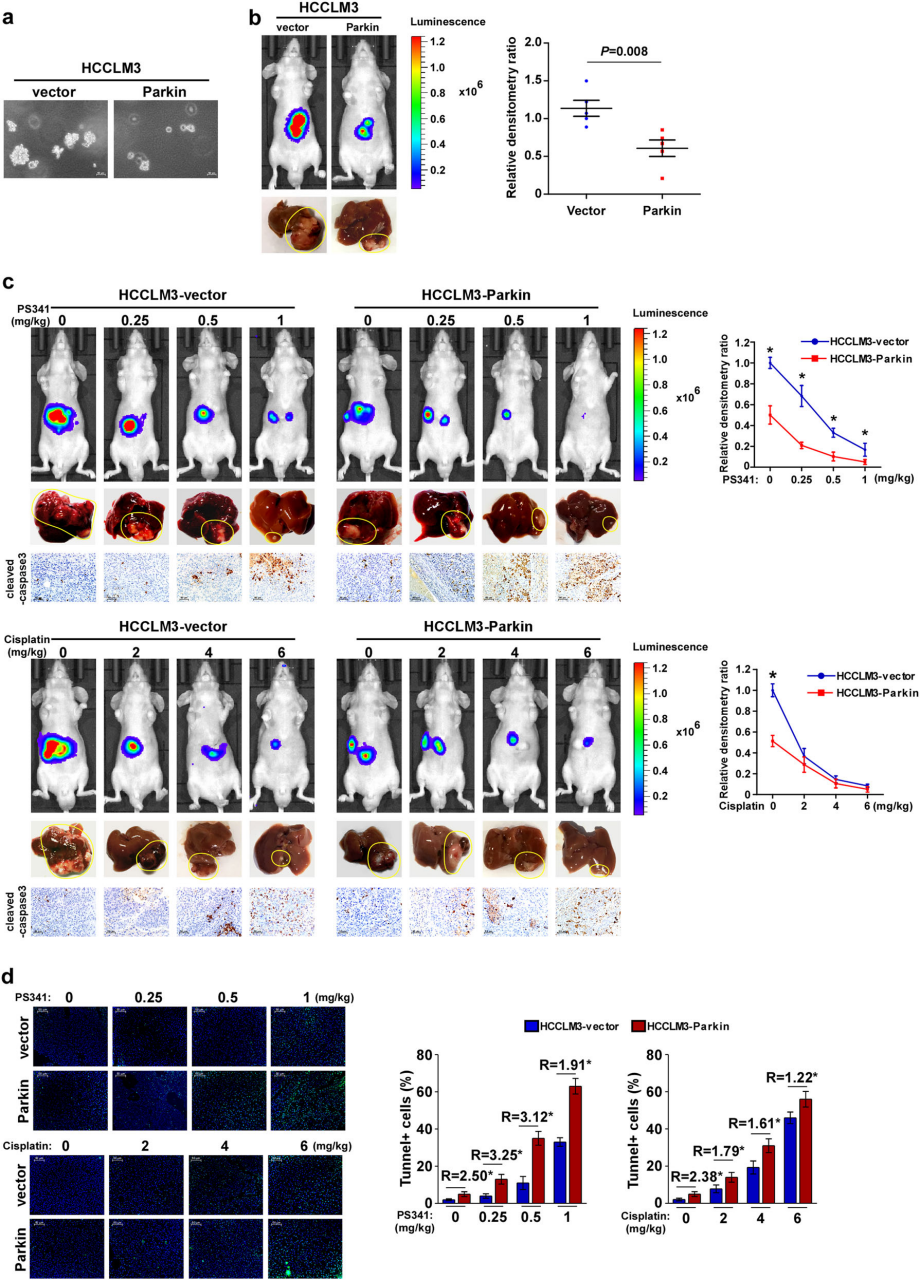
Parkin facilitates the PS341-induced cell apoptosis of HCC in vivo. **a** The tumorigenicity capability of indicated cells, determined by the soft agar clonogenic assay. Colonies larger than 0.1 mm in diameter were scored. **b** Bioluminescence images of orthotopic tumors. The relative densitometry ratios determined by bioluminescence imaging system software are shown on the right panel. **c** Bioluminescence images of orthotopic tumors showed that Parkin facilitates the PS341-inducing and cisplatin-inducing cell apoptosis in a dose-dependent manner. Bright-field images of livers and IHC analysis of cleaved-caspase-3 are shown below. The relative densitometry ratios determined by bioluminescence imaging system software are shown on the right panel. **d** The images and quantification of apoptotic cells in the liver tissues, determined by Tunel assay. R (ratio), the proportion of tunnel-positive cells in Parkin-overexpressing cells compared with that in vector control cells. The effect of Parkin on PS341-treated animals was more significant than that on cisplatin-treated animals. Students’ *t*-tests were used to compare the results of two groups, the vector and parkin-overexpressing groups. Error bar represents the mean ± SD of three independent experiments. *P < 0.05

Gene set enrichment analysis (GSEA) showed that Parkin expression correlated negatively with gene signatures related to cell proliferation, whereas it correlated positively to the caspase pathway and apoptosis process by using the TCGA HCC dataset (Fig. S3a). To further explore the biological function of Parkin in HCC, an in vivo orthotopic murine model was used. HCCLM3 cell lines exhibited a lower Parkin expression. We first generated the stable Parkin-overexpressed HCCLM3 cell line and its control (Fig. S3b). The soft agar clonogenic assay showed that the capacity of tumorigenicity of HCCLM3 cells was remarkably suppressed by Parkin overexpression (Fig. 2a). An orthotopic tumor model was performed by implanting Parkin-overexpressed HCC cells in the livers of nude mice. Notably, the tumor formation by Parkin-overexpressed HCCLM3 cells was smaller compared with the control group (Fig. 2b). These findings indicate that Parkin suppresses tumor growth in HCC cells in vivo.

We next sought to determine the effect of Parkin on tumor therapy in HCC cells in vivo. We compared the anticancer activity of the proteasome inhibitor PS341 as well as the classic chemotherapy drug cisplatin. Overexpression of Parkin enhanced these anticancer agents (especially PS341)-induced apoptotic cell death of HCC cells in vivo (Fig. 2c). The cleaved-caspase-3 expression (an important event of apoptosis) and the proportion of tunnel-positive cells were increased in Parkin-overexpressing cells compared with the control group (Fig. 2c, d). Together, these findings suggested that overexpression of Parkin enhances the chemotherapeutic agent-induced tumor suppression and apoptosis of HCC in vivo.

### Parkin facilitates the proteasome inhibitor-induced cell apoptosis of HCC in vitro

**Fig. 3:**
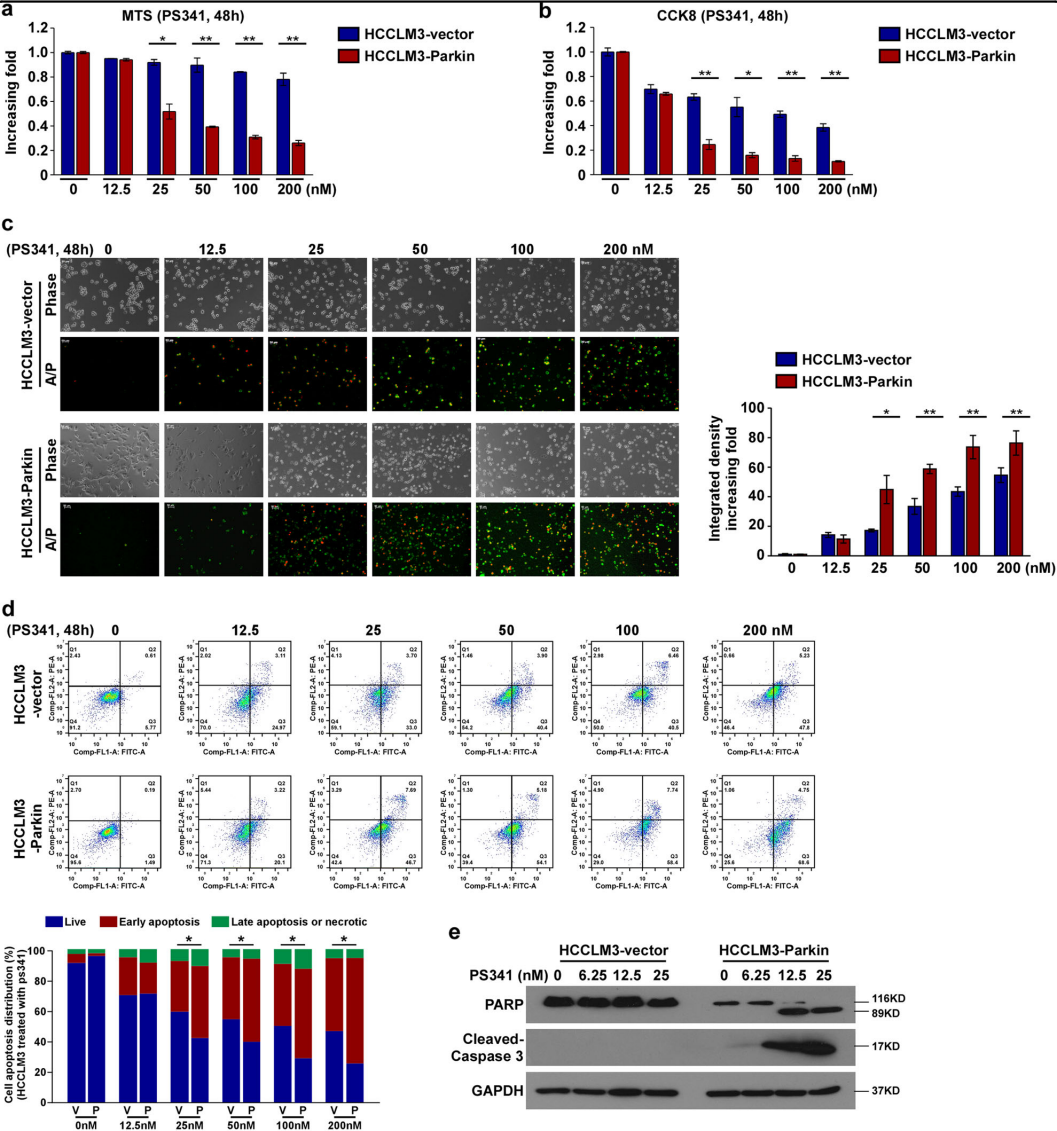
Parkin facilitates the proteasome inhibitor-induced cell apoptosis of HCC in vitro. **a**, **b** The cell viability of indicated cells measured by the MTS assay (**a**) and the CCK-8 assay (**b**). **c** The immunofluorescence staining of Annexin V-FITC (A) and PI (P) in indicated cells. **d** The flow-cytometry analysis of cell death in indicated cells. **e** Western blot analysis of apoptosis-related proteins in indicated cells. The indicated stable cell lines were used and treated with PS341 (48 h) in a dose-dependent manner. HCCLM3-vector and HCCLM3-Parkin stable cell lines were used in the experiments. A two-tailed *t*-test was used for the statistical analysis. Error bar represents the mean ± SD of three independent experiments. *P < 0.05, **P < 0.01

To explore the mechanism of action of Parkin on proteasome inhibitor-induced apoptosis, we generated Parkin-overexpressed or Parkin-knockdown HCC cell line. Compared with HCCLM3, the HepG2 cell exhibited a higher Parkin expression at the baseline. In addition to the overexpression of Parkin in the HCCLM3 cell line, we suppressed Parkin expression by RNAi in HepG2 cell lines (HepG2–Parkin–RNAi, Fig. S3b). These Parkin-associated genetic HCC cell lines were treated with the proteasome inhibitor PS341. The MTS assay revealed that the ectopic expression of Parkin increased PS341-induced growth inhibition in a dose-dependent manner (Figs. 3a and S4a). The CCK-8 assay further demonstrated that overexpression of Parkin decreased the cell viability following treatment with PS341 (Figs. 3b and S4b). Both the Annexin V/PI staining and the flow-cytometry analysis showed that Parkin increased PS341-inducing cell death, including apoptosis and necrosis, in a dose-dependent manner (Figs. 3c, d and S4c, d). Western blot analysis further found that PS341 induced the cleavage of PARP, and caspase-3 was enhanced by overexpression of Parkin, whereas it was inhibited by knockdown of Parkin (Figs. 3e and S4e), indicating that Parkin may play a major role in the induction of apoptosis. Parkin-mediated apoptosis activation was further confirmed by the treatment of the proteasome inhibitor MG132 (Figs. S5 and S6). In contrast, the function of Parkin in the regulation of apoptosis in HCC cell lines was not observed in LO2 cells (the normal human liver cell line) (Fig. S7a–c). Collectively, these data suggested that targeting Parkin only affects apoptosis in tumor cells, but not that in normal cells.

### Parkin inhibits NF-κB pathway activity

**Fig. 4:**
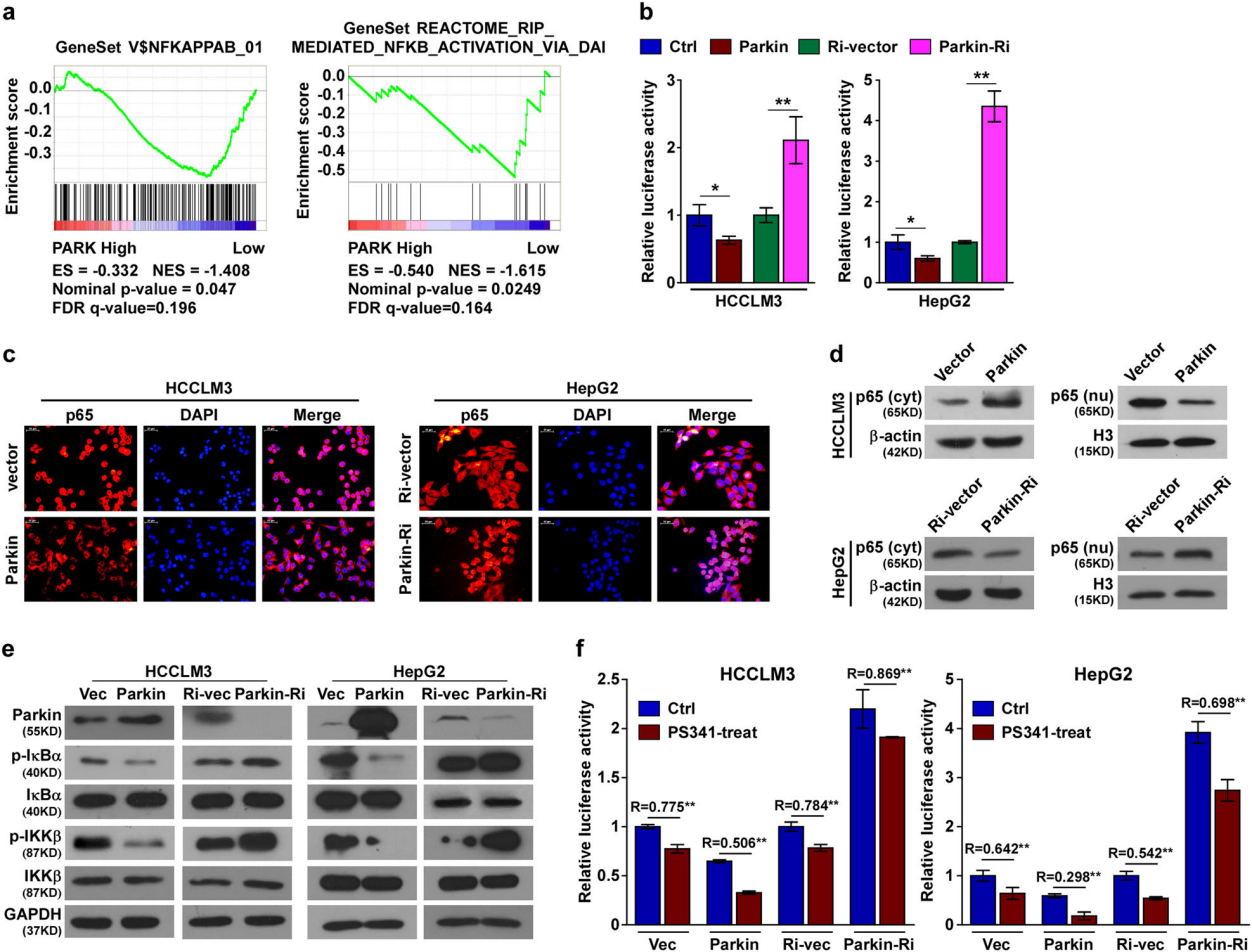
Parkin inhibits NF-κB signaling activity. **a** GSEA analysis showed that Parkin expression correlated negatively with NF-κB signaling. ES enrichment score, NES normalized enrichment score, FDR false discovery rate. **b** NF-κB luciferase reporter activity was analyzed in the indicated cells. **c** and **d** Subcellular localization of NF-κB/p65 in the indicated cells, as analyzed by immunofluorescence staining (**c**) and western blot analysis (**d**). nu nucleus, cyt cytoplasm. **e** Western blot analysis of NF-κB signaling pathway-related regulatory proteins. **f** NF-κB luciferase reporter activity was analyzed in the indicated cells. R (ratio), the relative NF-κB luciferase reporter activity in PS341-treated cells compared with that in control cells. The NF-κB activity inhibition of PS341 was more significant in Parkin-overexpressing cells, while it was attenuated in Parkin-silencing cells, than that in the control cells. Error bar represents the mean ± SD of three independent experiments. Ctrl control, Ri RNAi. *P < 0.05, **P < 0.01. A two-tailed *t*-test was used for the statistical analysis

We next define the mechanism of Parkin-mediated apoptosis in HCC cells. The analysis of GSEA revealed that *Parkin* mRNA expression correlated negatively with NF-κB activation (Fig. 4a), an important antiapoptotic mechanism in cancer therapy. To determine whether Parkin promotes apoptosis through control of the NF-κB pathway, we first analyzed NF-κB activation by the luciferase reporter assay. Indeed, the overexpression of Parkin blocked NF-κB luciferase activity, whereas knockdown of Parkin enhanced NF-κB luciferase activity (Fig. 4b). Furthermore, the analyses of NF-κB activation events including NF-κB nuclear translocation (Fig. 4c, d) and the expression of phosphorylated IκBα and phosphorylated IKKβ (Fig. 4e) confirmed that Parkin is a negative regulator of NF-κB activation.

Moreover, the inhibition of NF-κB activity by PS341 was found to be more dramatic in Parkin-overexpressed HCC cells, whereas PS341-induced NF-κB inhibition was attenuated by downregulation of Parkin (Fig. 4f). These findings suggested that Parkin facilitates the PS341-mediated NF-κB inhibition during apoptosis in HCC cells.

### Parkin inhibits NF-κB activation via direct degradation of TRAF2 and TRAF6

**Fig. 5:**
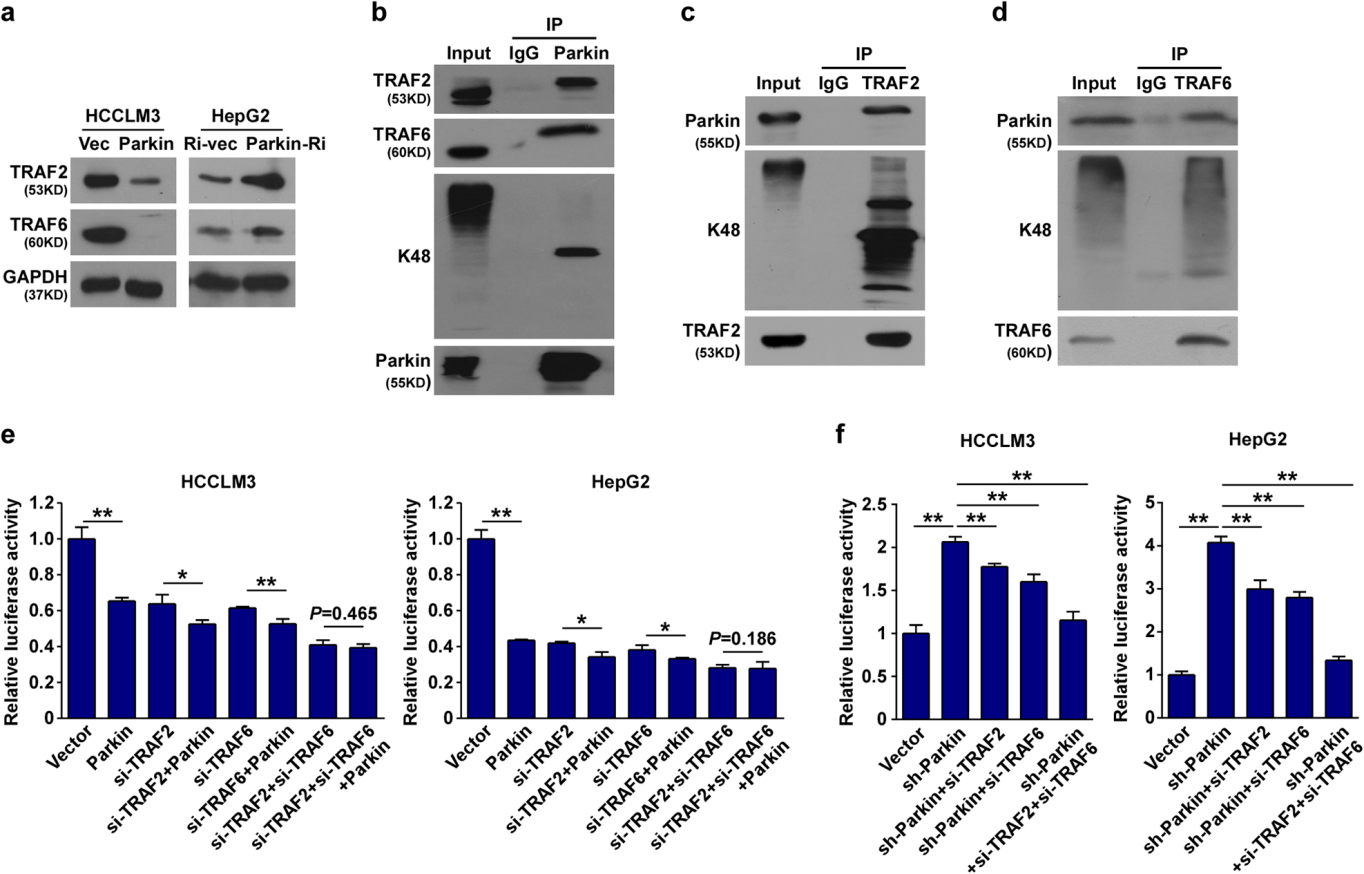
Parkin directly interacts with and leads to degradation of TRAF2 and TRAF6. **a** Western blot analysis of TRAF2 and TRAF6 in indicated cells. **b–d** IP analysis showing that Parkin interacts with TRAF2, TRAF6, and K48. Antibodies against Parkin, TRAF2, TRAF6, and K48 were used to perform co-IP. **e**, **f** NF-κB luciferase reporter activity was analyzed in the indicated cells. Vec vector, Ri RNAi. Error bar represents the mean ± SD of three independent experiments. *P < 0.05, **P < 0.01. A two-tailed *t*-test was used for the statistical analysis

We further investigated the molecular mechanism of Parkin-mediated NF-κB inhibition. As the phosphorylation of IκBα and IKKβ was downregulated by Parkin, the upstream regulators of the NF-κB signaling pathway, such as TRAF2 and TRAF6, were further chosen for examination. Western blot analysis revealed that the expressions of TRAF2 and TRAF6 were downregulated by ectopic Parkin and upregulated by Parkin inhibition (Fig. 5a). Co-IP analysis showed that both TRAF2 and TRAF6 interacted with Parkin and the K48-linked polyubiquitin (Fig. 5b–d), indicating that Parkin mediated TRAF2 and TRAF6 ubiquitin degradation. Like upregulation of Parkin, knockdown of TRAF2 or TRAF6 also diminished NF-κB luciferase activity (Fig. 5e). Of note, the enhanced NF-κB luciferase activity by silencing Parkin was attenuated by inhibition of TRAF2 or TRAF6 (Fig. 5f). These findings indicate that the Parkin-mediated NF-κB inhibition requires Parkin-mediated degeneration of TRAF2 and TRAF6.

**Fig. 6:**
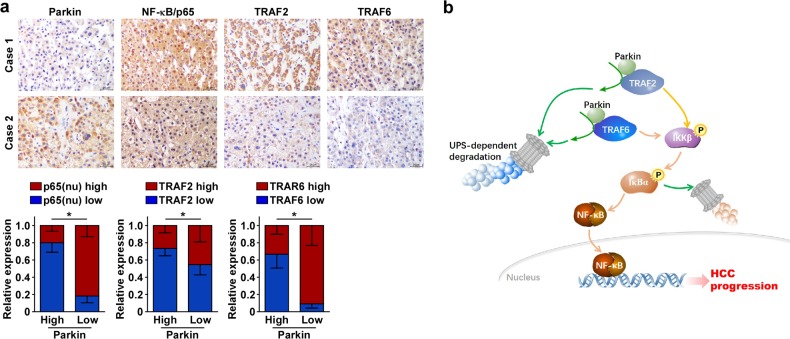
Parkin induced the downregulation of TRAF2 and TRAF6 and led to the inhibition of NF-κB in the clinic. **a** The higher expression levels of nuclear p65, TRAF2, and TRAF6 were associated with the lower expression of Parkin in HCC specimens determined by IHC analysis. Two representative cases are shown, magnification, ×400. nu nucleus. Error bar represents the mean ± SD of three independent experiments. *P < 0.05. A two-tailed *t*-test was used for the statistical analysis. **b** The schematic model of how Parkin mediates the inhibition of NF-κB and HCC progression

To further investigate the relationship between Parkin, NF-κB, TRAF2, and TRAF6, we analyzed the expression of these proteins in patients with HCC. IHC analysis of HCC specimens showed that higher expression of nuclear NF-κB (p65), TRAF2, and TRAF6 was strongly associated with a lower Parkin expression level (Fig. 6a). These findings further suggest that an abnormal expression of Parkin is related to the dysfunction of the NF-κB pathway.

## Discussion

HCC is one of the deadliest cancers due to its complexities and the poorest prognosis related to its high recurrence rate after surgical resection and the multidrug-resistance phenotype. It is characterized by late detection, fast progression, and poor response to therapy, underscoring the high mortality rate of this aggressive disease^33^. The present study demonstrated that Parkin plays a tumor-suppressor role in HCC and the downregulation of Parkin in HCC correlated with poor patient survival. Parkin is an important positive regulator of the anticancer activity of the proteasome inhibitor PS341 in vitro and in vivo. As an E3 proteasome ligase, Parkin can lead to TRAF2 and TRAF6 degradation through the protein–protein interaction, which blocks constitutive NF-κB activation and then triggers caspase-dependent apoptosis during PS341 treatment (Fig. 6b).

The UPS has emerged as a therapeutic target for various human diseases, including neurodegenerative diseases and cancer. Proteasome inhibition results in the buildup of misfolded and damaged intracellular proteins, leading to the disruption of multiple cellular signaling pathways and ultimately cell death^34^. However, the pharmacokinetic and pharmacodynamic characteristics and dose-limiting toxicities may limit the application of proteasome inhibitors in the treatment of solid tumors^8,12^. It has been reported that the aggregates of ubiquitin-conjugated protein (aggresome) formation block bortezomib-induced apoptosis in pancreatic cancer cells, and inhibition of the formation of the aggresome by the HDAC inhibitor can strongly potentiate the efficacy of bortezomib^35^. Combined with MD5-1, a tumor necrosis factor-related apoptosis-inducing ligand receptor agonist monoclonal antibody, bortezomib may be effective for metastatic solid tumor treatment through induction of apoptosis^36^. In addition, bortezomib can induce the expression of BIM, a member of the proapoptotic BH3-only protein, to suppress H-ras/MAPK pathway-dependent drug resistance^37^. In the present study, we demonstrated that overexpression of Parkin could enhance PS341-induced and MG132-induced apoptosis in HCC cells, indicating a new approach to overcome the drug resistance of proteasome inhibitors.

The NF-κB signaling pathway is constitutively activated in multiple cancers and is involved in tumorigenesis and metastasis^13^. UPS plays a critical role in the regulation of NF-κB activation in a context-dependent manner^38^. Proteasome inhibitors can inhibit the activation of NF-κB by blocking 26S proteasome-mediated IκB degradation^3,39^. USP4 has been demonstrated to suppress tumor migration and induction of apoptosis through interacting and deubiquitinating multiple NF-κB pathway-associated molecules (e.g., TRAF2, TRAF6, TAK1, and RIP)^40,41^. USP11 downregulates TNFα-mediated NF-κB activation through maintainance of the IκBα stability by its deubiquitination^42^. The COP9 signalosome regulates the assembly and activity of calling-RING ubiquitin ligases, which is involved in the ubiquitination of IκBα during NF-κB activation^43^. In contrast, we demonstrate that Parkin blocks NF-κB activation mainly through direct degradation of TRAF2 and TRAF6. Consistent with previous reports, as an E3 ligase, PARK2 has a selectivity for ubiquitin modification^44–46^, which is worth further investigation. Parkin itself can be ubiquitinated^47^, indicating a feedback between Parkin and UPS to control NF-κB activation.

The toxicity is an obstacle to proteasome inhibitors in the treatment of tumors^48^. It underscores the need for less-toxic proteasome inhibitors or lower-dose usage to avoid the toxicity. Our study also highlights that overexpression of Parkin can reduce the dose use of proteasome inhibitors and enhance the anticancer activity of cisplatin, PS341 and MG132. The selectivity between malignant and nonmalignant cells is essential for the tumor treatment. Malignant plasma cells are thought to be even more exquisitely sensitive to proteasome inhibition than nonmalignant plasma cells. This selectivity might be explained by the constitutive activation of NF‑κB in myeloma cells^49^. We demonstrate that the enhancement of proteasome inhibitors induced by apoptosis by Parkin in HCC is not observed in the normal hepatic cells, supporting that targeting Parkin is a safe and effective anticancer strategy for HCC.

As an E3 ubiquitin ligase, the major function of Parkin is to ligate ubiquitin to lysine residues, which is a crucial post-translational modification involved in almost all the cellular pathways. Parkin displays multivalent protective roles in dopaminergic neurons, while the loss function of Parkin accounts for 50% of ARPD^22^, correlating with the pathogenesis of sporadic and familial PD^50,51^. Parkin dysfunction has also been implicated in other neurodegenerative diseases, including Alzheimer’s disease and amyotrophic lateral sclerosis^52^. An increasing evidence indicates that Parkin functions as a tumor suppressor and deletion of Parkin has been found in multiple malignancies^24–26^. Consistently, we found that Parkin was downregulated and functioned as a suppressor in HCC. Moreover, the analysis of the TCGA database suggests that the downregulation of Parkin in various cancers may be due to the loss of heterozygosity and copy number of Parkin. It has been reported that Parkin may adopt different mechanisms of activation in different cellular backgrounds. The phosphorylation of Parkin at Ser65 of the Ubl domain, such as that by PINK1, leads to activation of Parkin and increases its ubiquitin ligase activity^53^, whereas phosphorylation of Parkin by Cdk5, c-Abl, Casein kinase-1, Protein kinase A, or Protein kinase C leads to its inactivation^54^. Thereore, it will be prospective to further investigate the function and regulation of Parkin either in neurological or malignant diseases. Clarifying Parkin’s role in disease progression will be important for targeting Parkin for therapeutic intervention.

In conclusion, our current study reveals a direct molecular link between Parkin and protein degradation in the control of the NF-κB pathway and may provide a novel UPS-dependent strategy for the treatment of HCC by induction of apoptosis.

## Materials and methods

### Materials

The reagents used in this study and their sources were Proteasome inhibitor PS341 (Bortezomib) and MG132 were purchased from MedChem Express (Princeton, NJ), and all of them were dissolved in dimethyl sulfoxide (DMSO) and stored at −80 °C. The antibodies purchased were Anti-TRAF2 (4724S), anti-TRAF6 (8028S), anti-cleaved Caspase-3 (9664S), anti-IKKβ (8943S), anti-IκBα (7543S), anti-phospho-IκBα (2859P), anti-NF-κB/p65 (8242S), anti-β-Actin (3700S), anti-Histone 3 (4499S), anti-K48-linkage-specific polyubiquitin (12805S), anti-GAPDH (5174S), anti-Rabbit IgG (2729), anti-mouse IgG (7076) (Cell Signaling Technology, Beverly, MA), anti-Parkin (ab15494, ab77924), and anti-phospho-IKKβ (ab59195) (Abcam, Cambridge, MA). The MTS assay (Cell Titer 96 Aqueous One Solution reagent) was purchased from Promega Corporation (Promega, Madison, WI). The CCK-8 assay kit (Cell Counting Kit-8, Dojindo, Japan), PI, and Annexin V-FITC Kits were purchased from Keygen Company (Nanjing, China). Dynabeads antibody-coupling kit was purchased from Life Technologies (Waltham, MA).

### Tissue specimens

A tissue array (*No. HLivH180Su14*) including 90 pairs of clinical HCC samples compared with their paired adjacent non-tumor tissues, was used for the detection of Parkin expression (Outdo Biotech, Shanghai, China). The investigation was conducted in accordance with ethical standards and according to the tenets of the Declaration of Helsinki and national and international guidelines. Clinical and clinicopathological classification and stage were determined according to the American Joint Committee on Cancer criteria. For the clinical materials used in our study, prior patient consent and approval from the Institutional Research Ethics Committee of Taizhou hospital were obtained. Clinical information about the samples is shown in Table S1.

### Immunohistochemistry analysis

For immunohistochemistry (IHC) analysis, paraffin-embedded specimens were cut into 4-µm sections, deparaffinized with xylene, rehydrated, and then submerged in EDTA-containing antigen retrieval buffer and microwaved for antigenic retrieval, as previously described^55^. Bovine serum albumin solution (1%) was used to block nonspecific binding. The sections were then incubated with anti-Parkin (Abcam, Cambridge, MA) antibodies overnight at 4 °C. The tissue sections were treated with biotinylated anti-rabbit secondary antibody (Thermo Fisher Scientific, Waltham, MA), and this was followed by further incubation with the streptavidin–horseradish peroxidase complex (Thermo Fisher Scientific), immersion in 3,3ʹ-diaminobenzidine, counterstaining with 10% Mayer’s hematoxylin, dehydration, and mounting.

The stained tumor sections were examined and scored independently by two observers for positively stained tumor cells and the intensity of immunohistochemical signals. The mean optical density (MOD) value determined by *Image J pro* software and the staining index (SI) were used for quantitative analysis of the IHC results. The SI was calculated as the staining intensity score multiplied by the proportion of positive tumor cells. According to the proportion of positively stained tumor cells, the sections were scored as follows: 0, no positive tumor cells; 1, <10% positive tumor cells; 2, 10–50% positive tumor cells; 3, >50% positive tumor cells. The intensity of staining was graded according to the following criteria: 0, no staining; 1, weak staining (light yellow); 2, moderate staining (yellow brown); 3, strong staining (brown). We assessed the expression of the indicated protein in IHC-stained tumor sections based on the SI scores as 0, 1, 2, 3, 4, 6, and 9. Cutoff values (SI ≥ 4 was considered as high expression) were chosen on the basis of a measure of heterogeneity with the log-rank test, with respect to survival analysis.

### Cell lines and cell culture

HepG2, Hep3B, PLC/PRF/5, and 293FT cell lines were purchased from American Type Culture Collection (ATCC, Manassas, VA); LO2, HCCLM3, and Huh7 cells were purchased from Procell (Procell Life Science &Technology, Wuhan, China). LO2 cells were maintained in RPMI-1640 medium (Gibco, Grand Island, NY), and all the HCC cell lines were cultured in Dulbecco’s modified Eagle’s medium (DMEM) (Gibco), supplemented with 10% fetal bovine serum (FBS) (HyClone, Logan, UT) and 100 units of penicillin–streptomycin at 37 °C with 5% CO_2_ atmosphere in a humidified incubator.

### Plasmids, siRNA, and transfection

The PARK2 plasmid and the control vector were purchased from GeneCopoeia Inc. (EX-Q0218-Lv128, NM_004562.2, FulenGen, Guangzhou). Hepatoma carcinoma cells were transduced with lentivirus particles expressing a short-hairpin RNA (shRNA) targeting the PARK2 sequence (HG12092-G G06N02M21 Sino Biological Inc.). The TRAF2 plasmid was obtained from OriGene Technologies Inc. (RC208110, Beijing), the TRAF6 plasmid was purchased from GeneCopoeia Inc. (EX-Q70134, FulenGen, Guangzhou). The reporter plasmid for quantitatively detecting the transcriptional activity of NF-κB was obtained as described previously^56^. TRAF2-siRNA (sc29509) and TRAF6-siRNA (sc36717) were purchased from Santa Cruz Biotechnologies (Santa Cruz, CA). Retroviral production, infection, and selection were performed as described previously^57^. Stable cell lines expressing PARK2 or PARK2 shRNA were selected for 14 days by using puromycin 48 h after infection. Plasmids and siRNA were transfected by using Lipofectamine 2000 (Invitrogen, Carlsbad, CA, USA).

### Cell viability assay

The MTS assay (Cell Titer 96 Aqueous One Solution reagent, Promega) and CCK-8 assay (Cell Counting Kit-8, Dojindo, Japan) were used to test the cell viability as previously reported^58^. Briefly, 1 × 10^4^ cells were plated in each well with triplicates and treated with the indicated drugs. Three hours before the cell culture termination, 20 μl of the MTS assay reagent or 10 μl of the CCK-8 assay reagent was added to each well of the 96-well plate. The absorbance density at a wavelength of 490 nm (MTS), or 450 nm (CCK-8), was read on a plate reader (Varioskan Flash 3001, Thermo Fisher, Waltham, MA).

### Cell death assay and flow-cytometry analysis

Apoptosis was assessed by flow cytometry (BD AccuriTM C6, Becton Dickinson and Company, USA) by using the Annexin V-FITC/PI apoptosis detection kit (Keygen Biotechnology, Nanjing, China). Cultured cells with drug treatment were collected, washed twice with the PBS, and then incubated in the working solution (500 μl of binding buffer with 5 μl of Annexin V-FITC) in the dark for 15 min; 5 μl of PI was added just before analysis. In addition, Annexin V-FITC/PI staining was also performed as described but was done so in situ. An inverted fluorescence microscope equipped with a digital camera (Axio Obsever Z1, Zeiss, Jena, Germany) was used to image the double-stained cells.

### Soft agar clonogenic assay

The anchorage-independent growth ability of cells was determined by the soft agar clonogenic assay. Cells (1 × 10^3^) were trypsinized and suspended in 2 ml of complete medium plus 0.33% agar (Sigma, St. Louis, MO). The agar-cell mixture was plated on top of a bottom layer comprising a complete medium with 0.66% agar. After 12–14 days, colony sizes were measured by using an ocular micrometer. Colonies >0.1 mm in diameter were scored.

### Immunoprecipitation (IP) and western blot analysis

For IP analysis, dynabeads coupled with antibodies were prepared and then cell lysates were added, and the antibody–lysate mixtures were rotated at 4 °C for 1 h. Immunocomplexes separated from dynabeads were washed with lysis buffer and then suspended with SDS blue loading buffer. To detect ubiquitinated proteins, lysis was performed at 70 °C for 10 min^59^. Western blot was performed as previously described^60^. Equal quantities of protein were electrophoresed through a 10% SDS–PAGE. In brief, equal amounts of total protein extracts from cultured cells were fractionated by 12% SDS–PAGE and electrically transferred onto polyvinylidene difluoride (PVDF) membranes. The blots were blocked with 5% milk for 1 h. Primary antibodies and the appropriate horseradish peroxidase-conjugated secondary antibodies were used to detect the designated proteins. The bounded secondary antibodies on the PVDF membrane were reacted to the ECL detection reagents (Santa Cruz) and exposed to X-ray films (Kodak, Rochester, NY, USA).

### Immunofluorescence staining

The cells were plated on coverslips and harvested for 24 h, then they were washed with ice-cold PBS, and fixed with 4% paraformaldehyde for 15 min, after which the coverslips were blocked with 10% BSA for 30 min and incubated with the primary antibody (anti-P65, Cell Signaling) for 1 h at room temperature. After washing with PBS, the coverslips were incubated with a fluorescein isothiocyanate-conjugated goat anti-Rabbit secondary antibody (Jackson ImmunoResearch, West Grove, PA) for 30 min. Cell nuclei were counterstained with DAPI (5 ng/ml) for 10 min. After washing with PBS, the coverslips were mounted with an anti-fading reagent (Invitrogen) and stored in the dark until evaluation. Gray-level images were acquired under a laser-scanning microscope (Olympus).

### Nuclear protein extraction assay

The nuclear protein extraction assay was performed by using Nuclear and Cytoplasmic Extraction Reagents (78835, Thermo Fisher). Briefly, cells (5 × 10^6^) were collected, gently resuspended with 200 μl of Cytoplasmic Extraction Reagent I, and incubated on ice for 15 min; then they were added with 11 μl of ice-cold Cytoplasmic Extraction Reagent II and incubated on ice for 1 min. By centrifuging the homogenate for 5 min at maximum speed in a microcentrifuge (16,000 × *g*), immediately transfer the supernatant (cytoplasmic extract) to a clean prechilled tube. Then, suspend the insoluble (pellet) fraction produced in the previous step with 100 μl of ice-cold Nuclear Extraction Reagent, and vortex the homogenate for 10 s. The homogenate was placed on ice and vortexing continued for 15 s every 10 min, for a total of 40 min, and then it was centrifuged for 10 min at maximum speed (16,000 × *g*) at 4 °C. Immediately transfer the supernatant (nuclear extract) fraction to a clean prechilled tube and store the extracts at −80 °C until use.

### Luciferase reporter assays

Cells (6 × 10^5^) were seeded in triplicate in 24-well plates and allowed to settle for 24 h. Indicated plasmids of 200 ng (e.g., luciferase reporter plasmids or the control plasmid) plus 1 ng of PRL-TK-Renilla plasmid and 50 ng of NF-κB-luc plasmid, were transfected into cells by using the Lipofectamine 2000 reagent (Invitrogen) according to the manufacturer’s instruction. Forty-eight hours after transfection, luciferase and Renilla signals were measured by using the Dual Luciferase Reporter Assay Kit according to the manufacturer’ s instruction (E1960, Promega).

### Xenografted tumor model and tumor tissue staining

BALB/c-nude mice (male, 4–5 weeks of age, weighing 18–20 g) were purchased from the Center of Experimental Animals of Guangzhou University of Chinese Medicine. All the experimental procedures were approved by the Institutional Animal Care and Use Committee of Guangzhou Medicine University. The BALB/c nude mice were randomly divided into four groups. Four groups of mice were inoculated orthotopically with HCCLM3-luc-Parkin cells, HCCLM3-luc-Vector cells, HepG2-luc-RNAi-Vector cells, and HepG2-luc-Parkin-RNAi cells (3 × 10^6^) in the liver tissue. Images were captured by using an in vivo bioluminescence imaging system (Xenogen IVIS Spectrum). The mice were intraperitoneally injected with different doses of chemotherapy, and the images were recorded of the volume changes of in situ tumors. Thirty days after tumor implantation, the mice were killed. Liver sections were fixed in formalin and embedded in paraffin by using the routine method. Serial 6.0-μm sections were cut and subjected to hematoxylin and eosin (H&E) staining with Mayer’s hematoxylin solution, analyzed by using IHC with an anti-cleaved-caspase-3 antibody, or performed by using the Tunel assay.

### Statistical analysis

All experiments were performed at least thrice. Data were analyzed statistically by using Fisher’s exact test, log-rank test, chi-square test, and Student’s two-tailed *t*-test. Survival curves were plotted by using the Kaplan–Meier method and compared by the log-rank test. GraphPad Prism 6.0 software and Excel were used for statistical analysis. The analysis of Gene Set Enrichment Analysis (GSEA) was performed according to the manual of GSEA papers^61,62^. Data represent mean ± standard deviation (SD) and *P* ≤ 0.05 was considered statistically significant.

## Supplementary information

supplementary files
