# Supplementary material for: Parkin facilitates proteasome inhibitor-induced apoptosis via suppression of NF-κB activity in hepatocellular carcinoma
Source: Cell Death Dis. 2019 Sep 26;10(10):719. doi: 10.1038/s41419-019-1881-x (PMC6763437; doi:10.1038/s41419-019-1881-x)
Supplement: Supplementary file 1 — supplementary files [file 41419_2019_1881_MOESM1_ESM.pdf]

Supplementary Figure legends

Figure S1. Parkin is downregulated in HCC. (a) IHC analysis of Parkin expression in formalin-fixed paraffin-embedded HCC tissues (T), compared with the adjacent non-tumor tissues (Adj.) from the same patients. **(b)** Kaplan–Meier analysis of overall survival for patients in breast cancer data of Kaplan Meier-plotter (http://kmplot.com). **(c)** The mRNA and protein expression of Parkin in HCC cell lines compared to the normal liver cells LO2. Error bar represents the mean ± SD of three independent experiments. * $P < 0.05$. Two-tailed t-test was used for the statistical analysis.

Figure S2. Parkin is downregulated in TCGA database. (a) Analysis of *Parkin* CNV in the TCGA dataset. **(b)** Expression of *Parkin* corresponding to different CNV levels in the TCGA dataset. **(c)** Expression of *Parkin* in a subset of human tumors (T) from the TCGA dataset, in comparison with normal tissues (N). **(d)** The deletion rate of *Parkin* in a subset of human tumors from the TCGA dataset. ** $P < 0.01$. Two-tailed t-test was used for the statistical analysis.

Figure S3. Parkin correlates with proliferation inhibition and apoptosis process. (a) GSEA analysis showing that Parkin expression correlated negatively with proliferation, positively with caspase pathway and apoptosis processes. **(b)** Western blot analysis of Parkin expression in indicated cells. All the data were performed by three independent experiments.

Figure S4. Parkin facilitates the proteasome inhibitor induced cell apoptosis in HCC *in vitro*. (a-b) The cell viability of indicated cells measured by MTS assay (a) and CCK-8 assay (b). **(c)** The immunofluorescence staining of Annexin V-FITC (A) and PI (P) in indicated cells. **(d)** The flow cytometry analysis of cell death in indicated cells. **(e)** Western blot analysis of apoptosis related proteins in indicated cells. HepG2-RNAi-vector and HepG2-Parkin-RNAi stable cell lines, treated with PS341 (48h), were used in the experiments. Two-tailed *t*-test was used for the statistical analysis. Error bar represents the mean ± SD of three independent experiments. * $P < 0.05$, ** $P < 0.01$.

Figure S5. Parkin facilitates the proteasome inhibitor induced cell apoptosis in HCC *in vitro*. (a-b) The cell viability of indicated cells measured by MTS assay (a) and CCK-8 assay (b). **(c)** The immunofluorescence staining of Annexin V-FITC (A) and PI (P) in indicated cells. **(d)** The flow cytometry analysis of cell death in indicated

cells. **(e)** Western blot analysis of apoptosis related proteins in indicated cells. HCCLM3-vector and HCCLM3-Parkin stable cell lines, treated with MG132 (24h), were used in the experiments. Two-tailed *t*-test was used for the statistical analysis. Error bar represents the mean ± SD of three independent experiments. * $P < 0.05$, ** $P < 0.01$.

Figure S6. Parkin facilitates the proteasome inhibitor induced cell apoptosis in HCC *in vitro*. (a-b) The cell viability of indicated cells measured by MTS assay (a) and CCK-8 assay (b). **(c)** The immunofluorescence staining of Annexin V-FITC (A) and PI (P) in indicated cells. **(d)** The flow cytometry analysis of cell death in indicated cells. **(e)** Western blot analysis of apoptosis related proteins in indicated cells. HepG2-RNAi-vector and HepG2-Parkin-RNAi stable cell lines, treated with MG132 (24h), were used in the experiments. Two-tailed *t*-test was used for the statistical analysis. Error bar represents the mean ± SD of three independent experiments. * $P < 0.05$, ** $P < 0.01$.

Figure S7. Parkin shows no significant effect on the proteasome inhibitor-induced cell apoptosis in normal hepatic cells *in vitro*. (a) Western blot analysis of Parkin expression in indicated cells. **(b-c)** The cell viability of indicated cells measured by MTS assay (b) and CCK-8 assay (c). LO2-vector, LO2-Parkin and LO2-Parkin-RNAi stable cell lines were used and both treated with PS341 (48h) or MG132 (24h) in a dose-dependent manner. Error bar represents the mean ± SD of three independent experiments. Two-tailed t-test was used for the statistical analysis.

Table S1. Clinicopathological characteristics of studied patients and expression of Parkin in HCC

**Factor**	**No.**	**(%)**
**Age (years)**		
≤52	50	55.6%
>52	40	44.4%
**Gender**		
Male	80	88.9%
Female	10	11.1%
**Clinical stage**		
I	3	3.3%
II	40	44.4%
III	47	52.2%
**T**		
T1	58	64.5%
T2	28	31.1%
T3	4	4.44%
N		
N0	90	100%
N1	0	0%
M		
M0	90	100%
M1	0	0%
**Vital status**		
Alive	58	64.4%
Dead	32	35.6%
**Expression of Parkin (determined by MOD)**		
Low expression	45	50%

High expression	45	50%
**Expression of Parkin (determined by SI)**		
Low expression	67	74.4%
High expression	23	25.6%

Figure S1

a

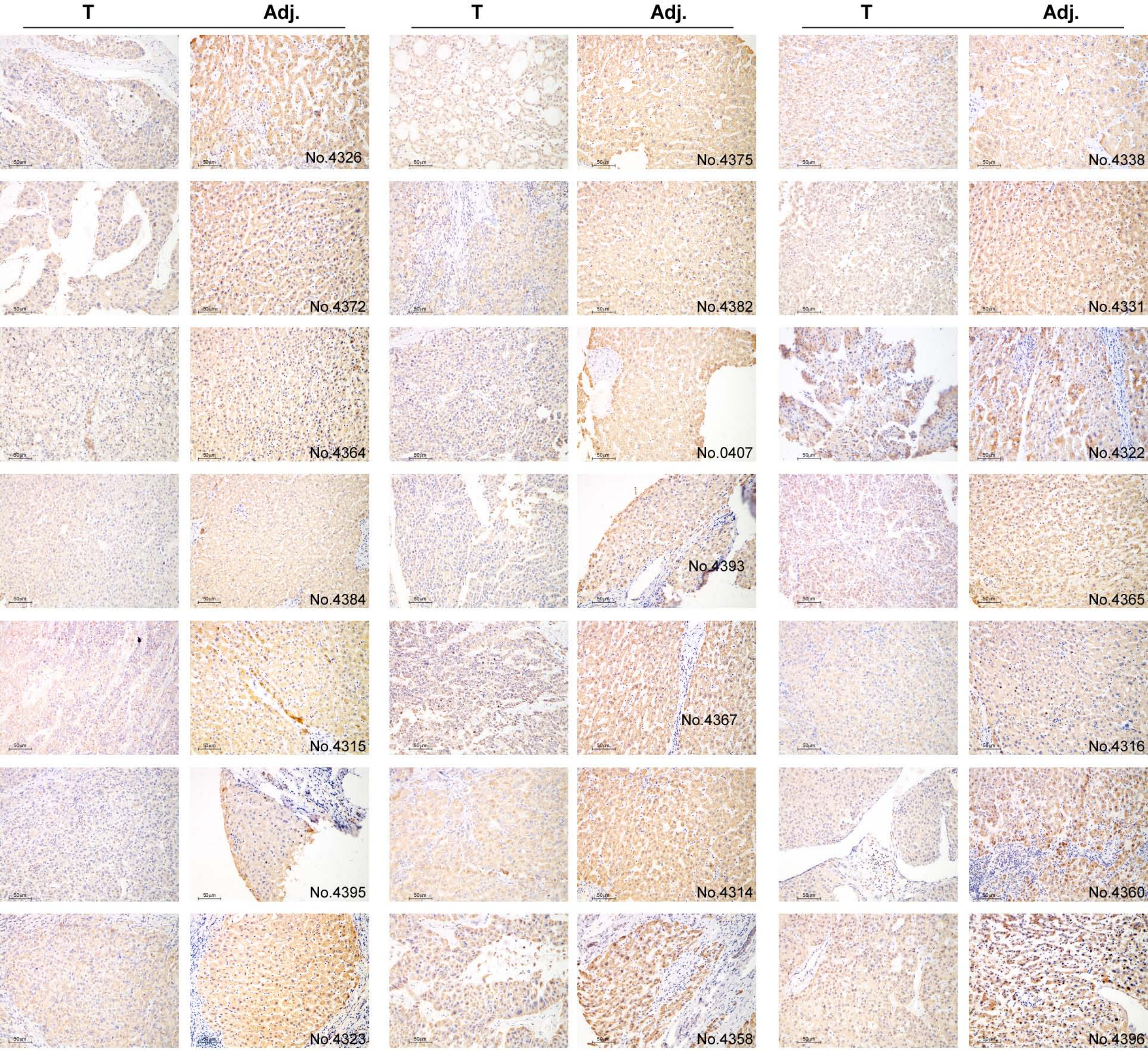

b

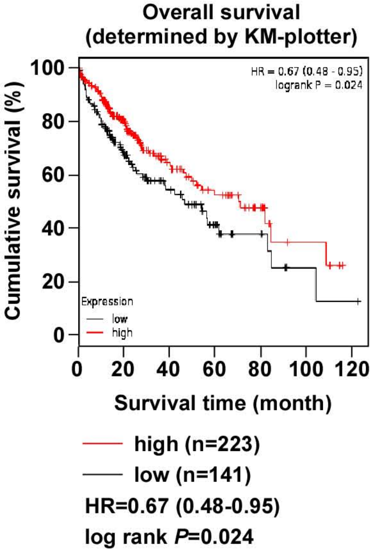

c

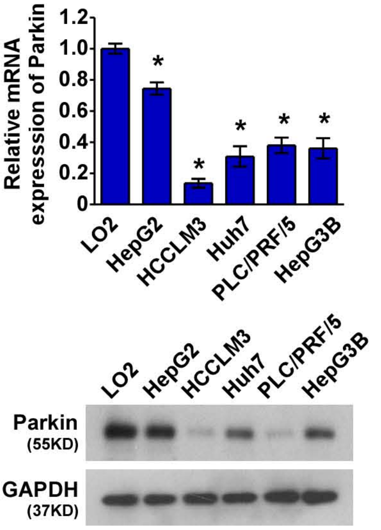

Figure S2

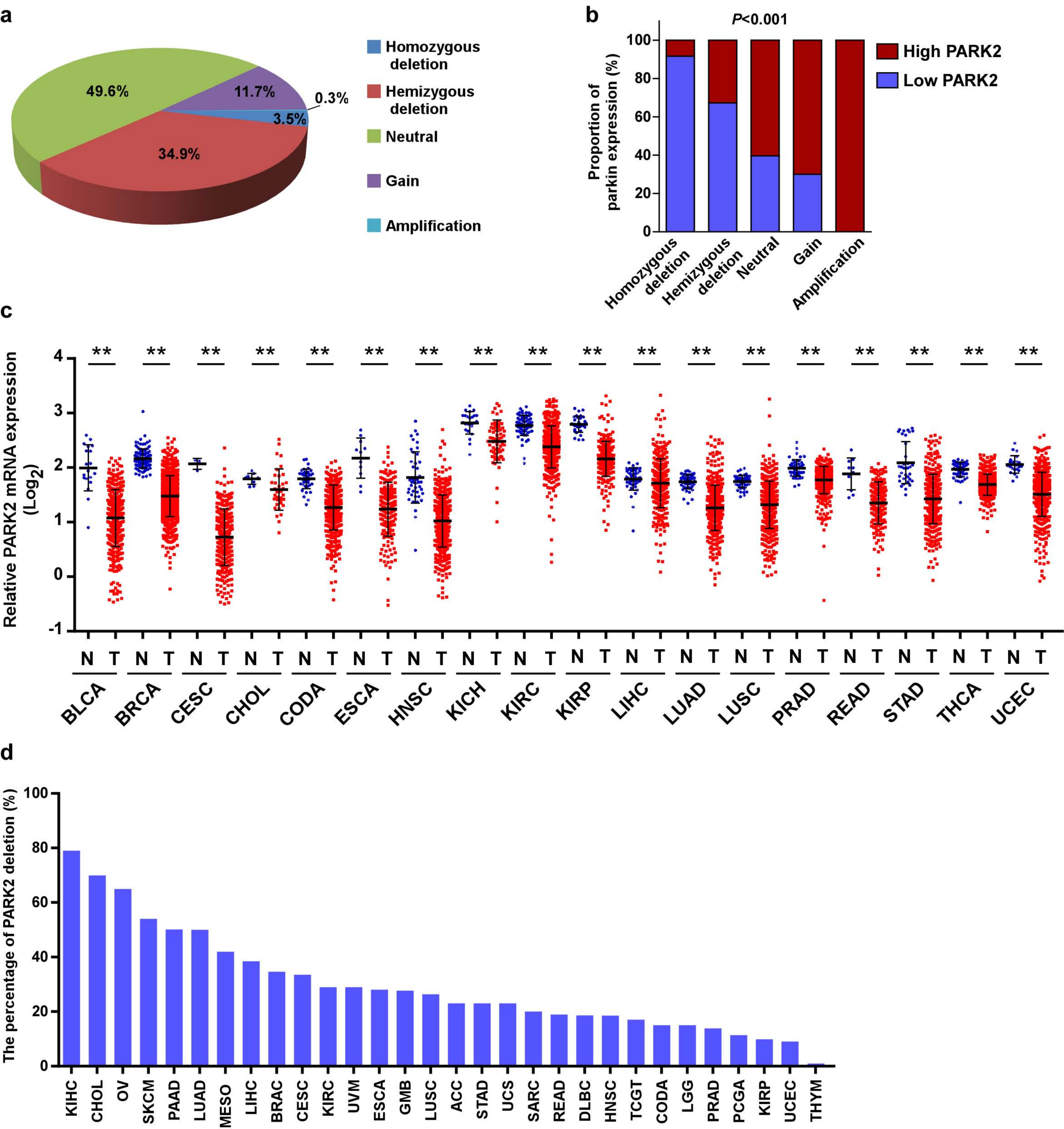
a
Homozygous deletion
Hemizygous deletion
Neutral
Gain
Amplification
49.6%
11.7%
0.3%
3.5%
34.9%
b
P<0.001
Proportion of parkin expression (%)
High PARK2
Low PARK2
Homozygous deletion
Hemizygous deletion
Neutral
Gain
Amplification
c
Relative PARK2 mRNA expression (Log2)
N T
BLCA BRCA CESC CHOL CODA ESCA HNSC KICH KIRC KIRP LIHC LUAD LUSC PRAD READ STAD THCA UCEC
d
The percentage of PARK2 deletion (%)
KIHC CHOL OV SKCM PAAD LUAD MESO LIHC BRAC CESC KIRC UVM ESCA GMB LUSC ACC STAD UCS SARC READ DLBC HNSC TCGT CODA LGG PRAD PCGA KIRP UCEC THYM

Figure S3

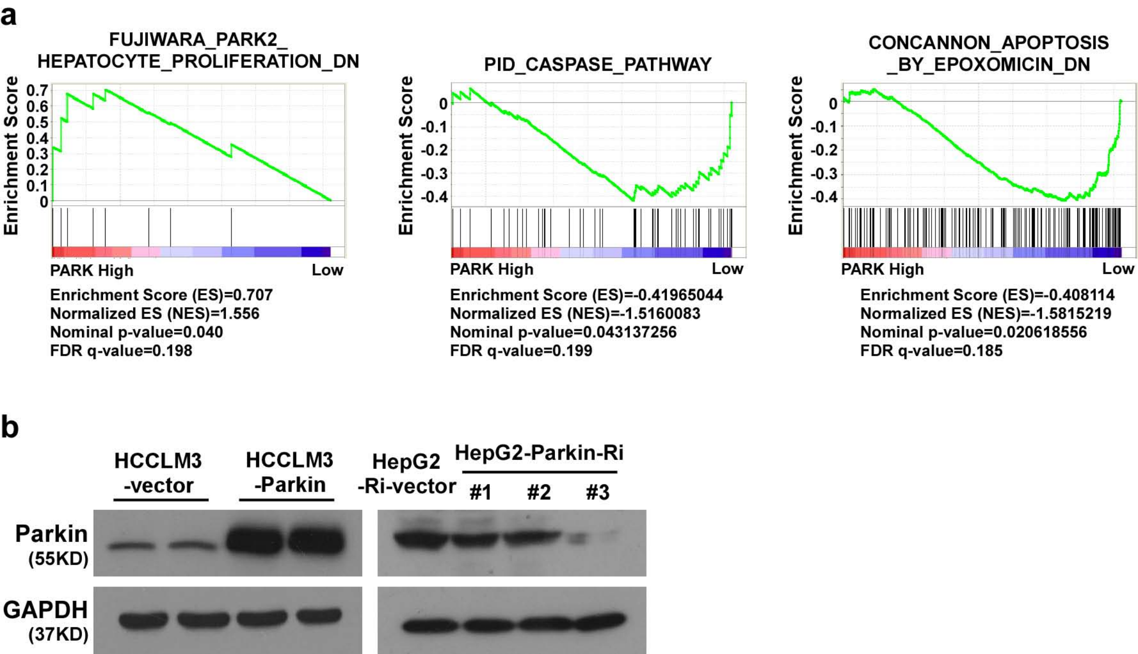
a
FUJIWARA_PARK2_
HEPATOCYTE_PROLIFERATION_DN
Enrichment Score
0.7
0.6
0.5
0.4
0.3
0.2
0.1
0
PARK High
Low
Enrichment Score (ES)=0.707
Normalized ES (NES)=1.556
Nominal p-value=0.040
FDR q-value=0.198
PID_CASPASE_PATHWAY
Enrichment Score
0
-0.1
-0.2
-0.3
-0.4
PARK High
Low
Enrichment Score (ES)=-0.41965044
Normalized ES (NES)=-1.5160083
Nominal p-value=0.043137256
FDR q-value=0.199
CONCANNON_APOPTOSIS
_BY_EPOXOMICIN_DN
Enrichment Score
0
-0.1
-0.2
-0.3
-0.4
PARK High
Low
Enrichment Score (ES)=-0.408114
Normalized ES (NES)=-1.5815219
Nominal p-value=0.020618556
FDR q-value=0.185
b
HCCLM3
-vector
HCCLM3
-Parkin
HepG2
-Ri-vector
HepG2-Parkin-Ri
#1
#2
#3
Parkin
(55KD)
GAPDH
(37KD)

Figure S4

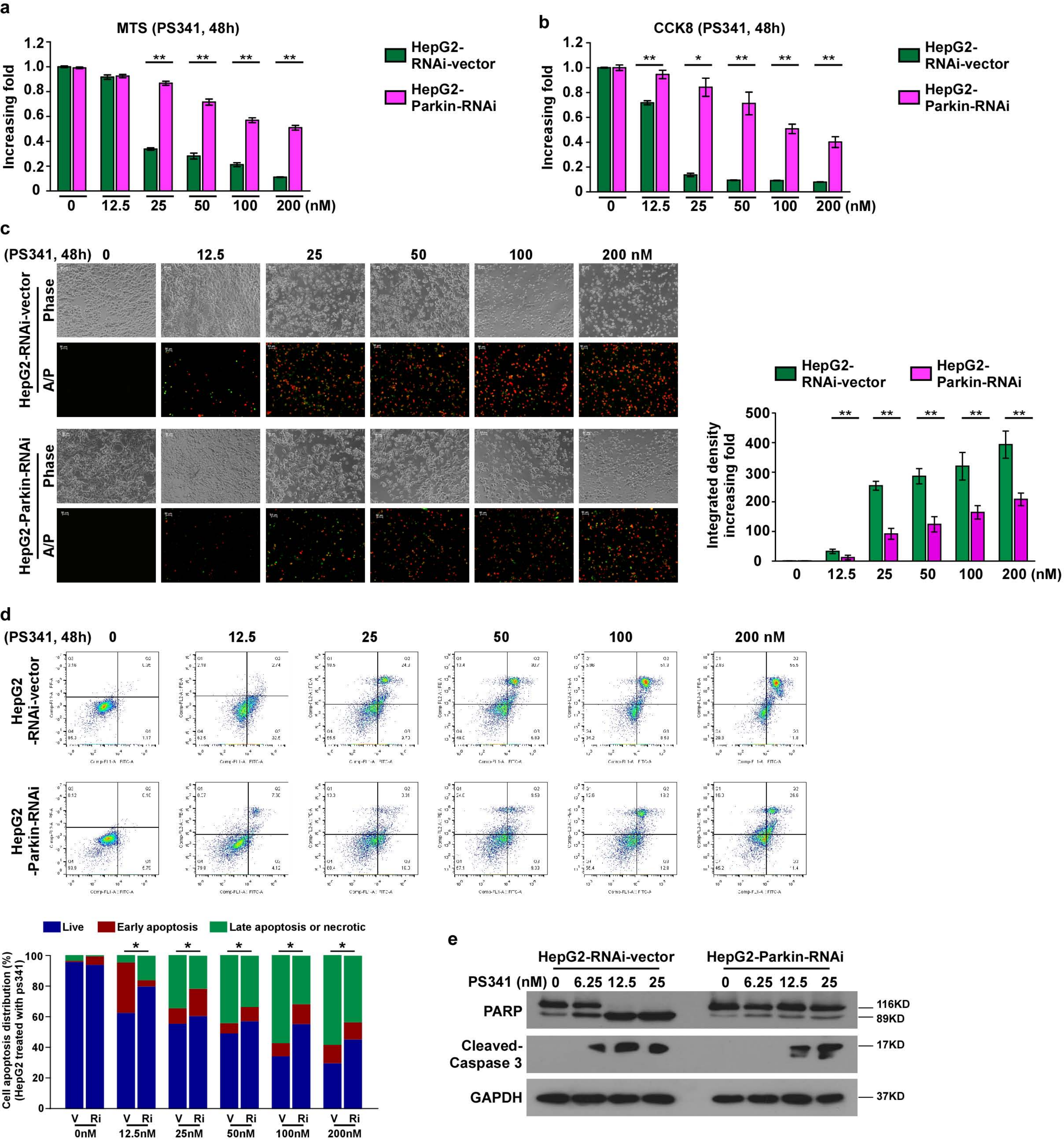
a
MTS (PS341, 48h)
Increasing fold
0 12.5 25 50 100 200 (nM)
HepG2-RNAi-vector
HepG2-Parkin-RNAi
b
CCK8 (PS341, 48h)
Increasing fold
0 12.5 25 50 100 200 (nM)
HepG2-RNAi-vector
HepG2-Parkin-RNAi
c
(PS341, 48h) 0 12.5 25 50 100 200 nM
HepG2-RNAi-vector
Phase
A/P
HepG2-Parkin-RNAi
Phase
A/P
HepG2-RNAi-vector
HepG2-Parkin-RNAi
Integrated density increasing fold
0 12.5 25 50 100 200 (nM)
d
(PS341, 48h) 0 12.5 25 50 100 200 nM
HepG2 -RNAi-vector
HepG2 -Parkin-RNAi
Live
Early apoptosis
Late apoptosis or necrotic
Cell apoptosis distribution (%) (HepG2 treated with ps341)
V Ri
0nM 12.5nM 25nM 50nM 100nM 200nM
e
HepG2-RNAi-vector
HepG2-Parkin-RNAi
PS341 (nM) 0 6.25 12.5 25 0 6.25 12.5 25
PARP
Cleaved-Caspase 3
GAPDH
116KD
89KD
17KD
37KD

Figure S5

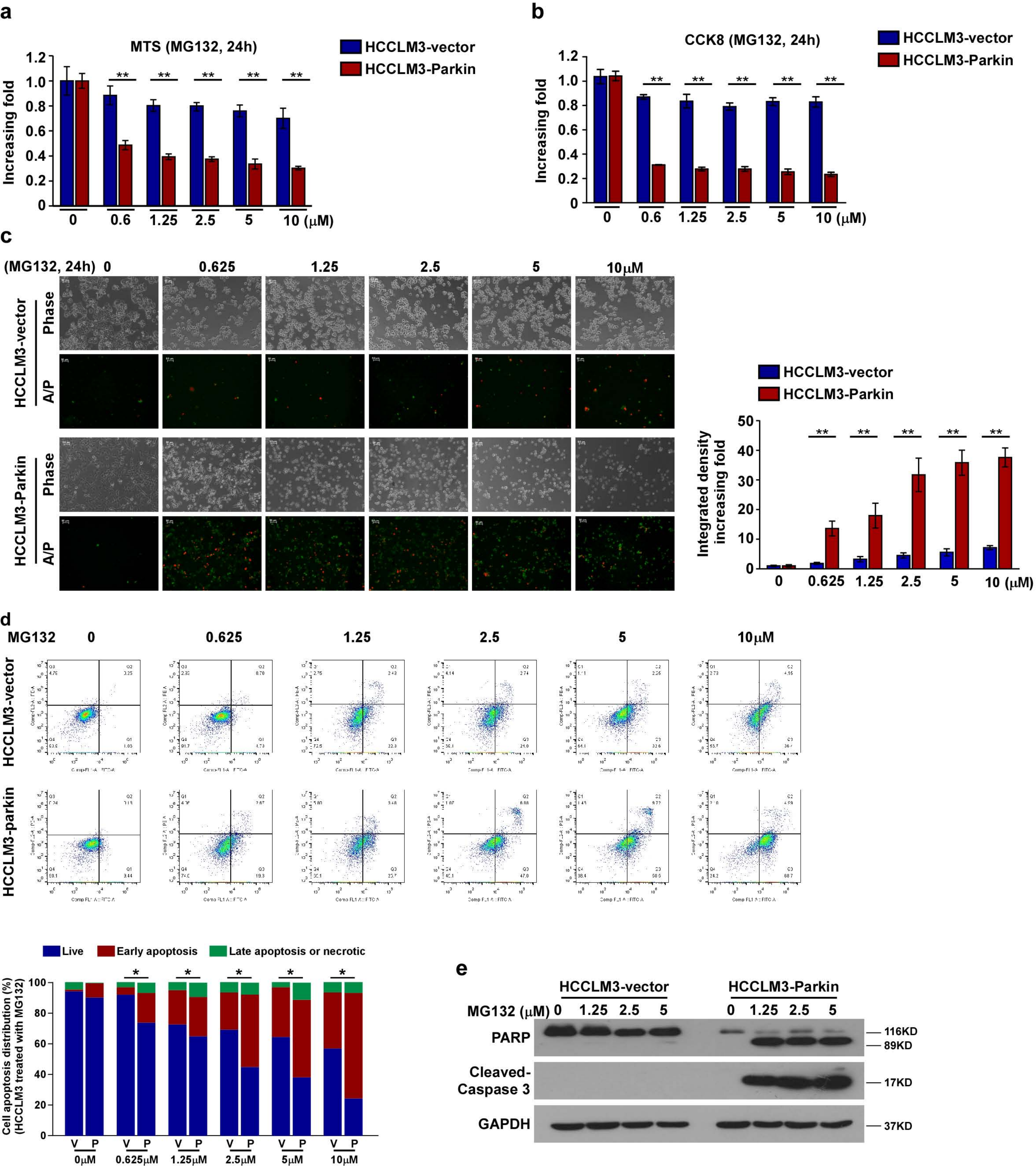
a
MTS (MG132, 24h)
HCCLM3-vector
HCCLM3-Parkin
Increasing fold
1.2
1.0
0.8
0.6
0.4
0.2
0
**
0
0.6
1.25
2.5
5
10 (μM)
b
CCK8 (MG132, 24h)
HCCLM3-vector
HCCLM3-Parkin
Increasing fold
0
0.6
1.25
2.5
5
10 (μM)
c
(MG132, 24h) 0
0.625
1.25
2.5
5
10μM
HCCLM3-vector
HCCLM3-Parkin
Phase
A/P
HCCLM3-vector
HCCLM3-Parkin
Integrated density increasing fold
50
40
30
20
10
0
0
0.625
1.25
2.5
5
10 (μM)
d
MG132
0
0.625
1.25
2.5
5
10μM
HCCLM3-vector
HCCLM3-parkin
Live
Early apoptosis
Late apoptosis or necrotic
Cell apoptosis distribution (%) (HCCLM3 treated with MG132)
100
80
60
40
20
0
*
V
P
0μM
0.625μM
1.25μM
2.5μM
5μM
10μM
e
HCCLM3-vector
HCCLM3-Parkin
MG132 (μM) 0
1.25
2.5
5
PARP
Cleaved-Caspase 3
GAPDH
116KD
89KD
17KD
37KD

Figure S6

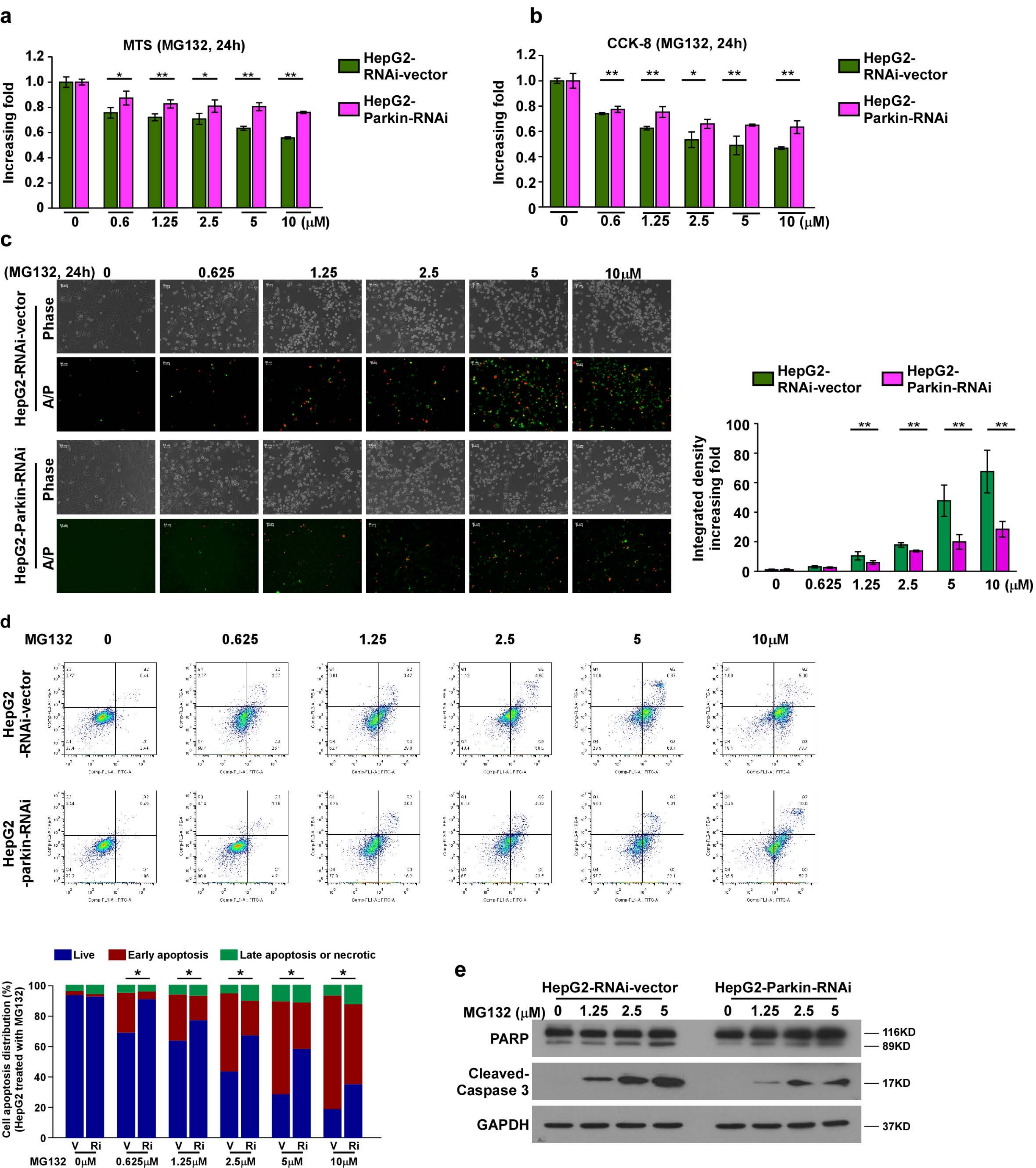
a
MTS (MG132, 24h)
Increasing fold
HepG2-RNAi-vector
HepG2-Parkin-RNAi
0 0.6 1.25 2.5 5 10 (μM)
b
CCK-8 (MG132, 24h)
Increasing fold
HepG2-RNAi-vector
HepG2-Parkin-RNAi
0 0.6 1.25 2.5 5 10 (μM)
c
(MG132, 24h) 0 0.625 1.25 2.5 5 10μM
HepG2-RNAi-vector
HepG2-Parkin-RNAi
Phase
A/P
Integrated density increasing fold
0 0.625 1.25 2.5 5 10 (μM)
d
MG132 0 0.625 1.25 2.5 5 10μM
HepG2 -RNAi-vector
HepG2 -parkin-RNAi
Live
Early apoptosis
Late apoptosis or necrotic
Cell apoptosis distribution (%) (HepG2 treated with MG132)
V Ri
MG132 0μM 0.625μM 1.25μM 2.5μM 5μM 10μM
e
HepG2-RNAi-vector
HepG2-Parkin-RNAi
MG132 (μM) 0 1.25 2.5 5 0 1.25 2.5 5
PARP
Cleaved-Caspase 3
GAPDH
116KD
89KD
17KD
37KD

Figure S7

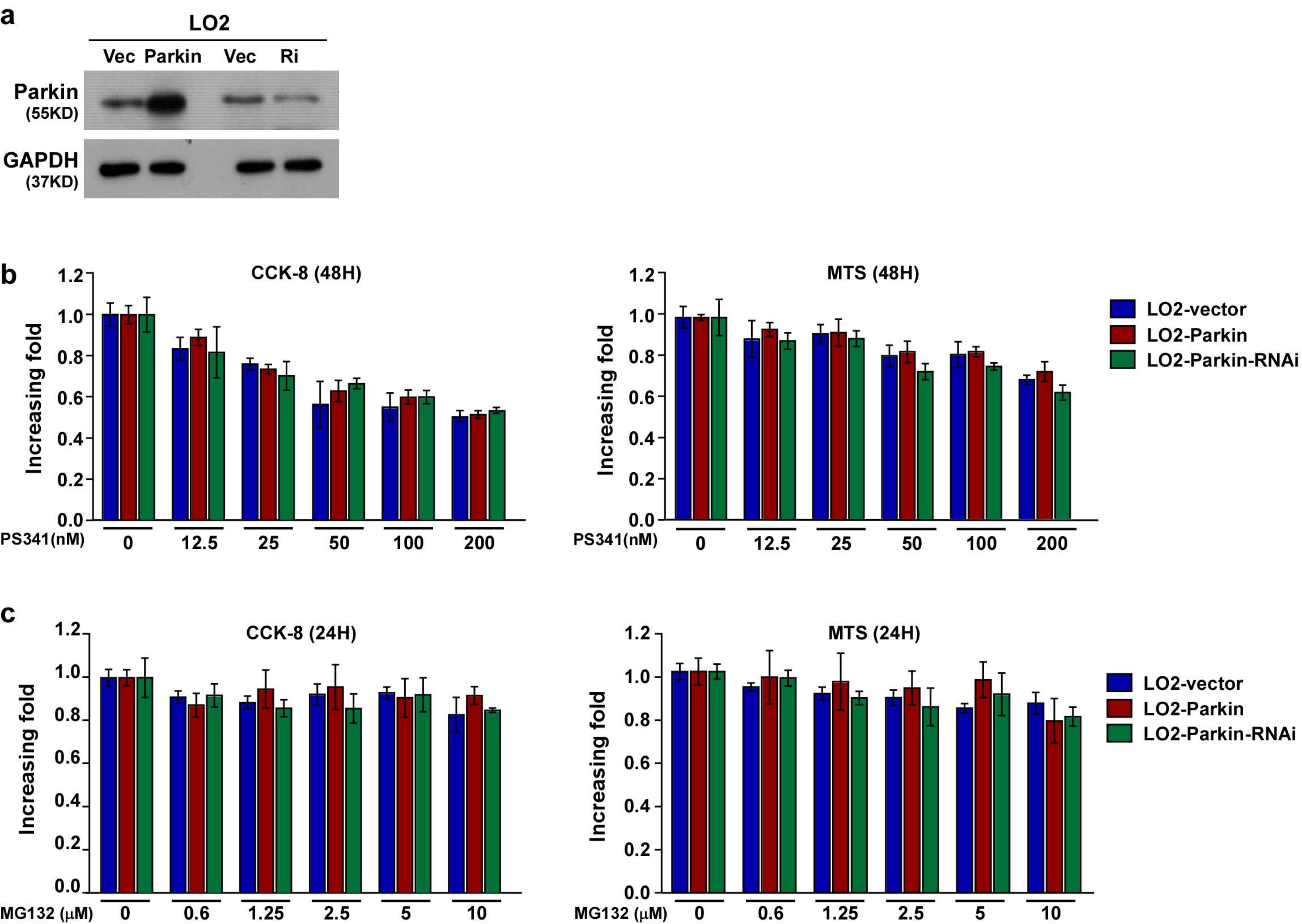
a
LO2
Vec
Parkin
Vec
Ri
Parkin
(55KD)
GAPDH
(37KD)
b
CCK-8 (48H)
MTS (48H)
Increasing fold
1.2
1.0
0.8
0.6
0.4
0.2
0.0
PS341(nM)
0
12.5
25
50
100
200
LO2-vector
LO2-Parkin
LO2-Parkin-RNAi
c
CCK-8 (24H)
MTS (24H)
MG132 (μM)
0
0.6
1.25
2.5
5
10
